# Influence of Aging and Immune Alterations on Susceptibility to Pneumococcal Pneumonia in the Elderly

**DOI:** 10.3390/pathogens14010041

**Published:** 2025-01-08

**Authors:** Nathan Kang, Veedamali S. Subramanian, Anshu Agrawal

**Affiliations:** 1 Division of Basic and Clinical Immunology, Department of Medicine, University of California Irvine, Irvine, CA 92697, USA; 2 Division of Gastroenterology, Department of Medicine, University of California Irvine, Irvine, CA 92697, USA; vsubrama@uci.edu

**Keywords:** aging, *Streptococcus pneumoniae*, immunity

## Abstract

Pneumonia is a common respiratory infection affecting individuals of all ages, with a significantly higher incidence among the elderly. As the aging population grows, pneumonia is expected to become an increasingly critical health concern. In non-institutionalized elderly individuals, the annual incidence ranges from 25 to 44 per 1000, approximately four times higher than in those under 65. *Streptococcus pneumoniae*, a Gram-positive diplococcus, is the leading cause of pneumonia-related deaths in older adults. Management of *S. pneumoniae* infections in the elderly is challenging due to impaired antibody responses to polysaccharides and surface proteins, compounded by rising antibiotic resistance. The underlying mechanisms for increased susceptibility remain unclear, but age-related changes in the immune system, particularly in dendritic cells and T cells, are implicated. This review explores how aging-related immune alterations contribute to the heightened vulnerability of the elderly to *S. pneumoniae* infections.

## 1. Introduction

*Streptococcus pneumoniae* (Pneumococcus) is a Gram-positive, catalase-negative species of bacterium responsible for most bacterial pneumonia cases globally and affects approximately 39% of individuals in the United States aged 65 and older with a chronic medical condition [1,2]. *S. pneumoniae* is commonly spread through respiratory droplets and colonizes in the upper respiratory tract. The ability of *S. pneumoniae* to spread through the air makes it one of the most commonly spread diseases in hospitals. Hospital-acquired pneumonia makes up approximately 15% of hospital-acquired infections in the United States, with some studies showing that approximately 12.1% of all community-acquired pneumonia involves the *S. pneumoniae* bacteria [1,2]. Older individuals are at a high risk of being infected with hospital-acquired pneumococcal disease since they make up a large percentage of hospital patients, and in 2018, it was estimated that approximately 16.8% of individuals over the age of 65 in the United States were hospitalized at least once that year [3,4]. The substantial percentage of hospitalizations for older individuals makes them more vulnerable to being affected by hospital-acquired pneumococcal disease and they often suffer more severe symptoms due to their weakened immune system.

## 2. Mechanism of Infection

**Figure 1:**
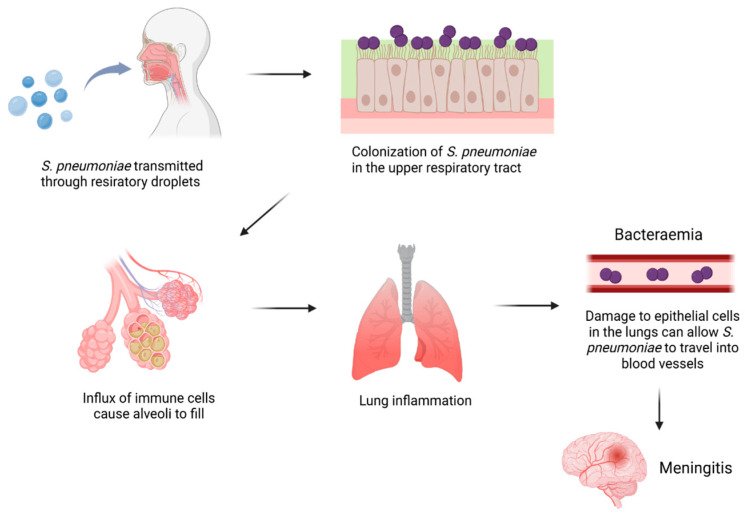
Pathogenesis of pneumococcal disease. Figure created using BioRender (https://www.biorender.com/, accessed on 11 November 2024).

*S. pneumoniae* is a spherical diplococci bacterium with thick peptidoglycan cell walls containing pneumococcal lipoteichoic acid (pnLTA), a polysaccharide that stimulates the production of inflammatory cytokines such as IL-6, TNF-α, and IFN-γ. The presence of the choline-binding protein A (CbpA) enables *S. pneumoniae* to bind to host cells in the nasopharynx through non-covalent interactions with choline residues on the cell walls. The colonization of pneumococcus in the upper respiratory tract leads to an influx of red and white blood cells that fill the air sacs [5,6,7,8,9,10]. Neutrophils recruited to the lungs undergo degranulation and release neutrophil elastase, a serine protease that facilitates phagocytosis and the destruction of pneumococcus bacteria. However, neutrophil elastase can break down elastin in the lung tissue, a vital structural protein, deteriorating the alveolar walls and impairing lung function. In addition, the neutrophils further recruit immune cells to the lungs due to the release of IL-8, TNF-α, and IL-1β, potent pro-inflammatory cytokines [11,12,13,14]. Excessive inflammation exacerbates tissue damage and can lead to the formation of scar tissue that can impair respiratory function and reduce oxygen levels in the blood (Figure 1). Older individuals often have weakened immune systems due to changes such as thymus involution, the natural shrinkage of the thymus that occurs with age, and the bone marrow producing fewer immune cells, which occurs due to the declining function of hematopoietic stem cells that occurs naturally with age [6]. Shrinkage in the thymus results in reduced T cell production, which makes it harder for the individual’s immune system to fight off pathogens. Declining immune cell production in the bone marrow can also lead to an increased risk of infection due to the smaller number of functional immune cells that can destroy pathogens [10]. The increased vulnerability of older individuals to the inflammatory effects brought about by *S. pneumoniae* contributes to the high mortality rate for patients over the age of 65, with the mortality rate due to *S. pneumoniae* among children under the age of 15 being approximately 4% while the mortality rate for individuals over the age of 65 is about 21% [15].

*S. pneumoniae* contains many virulent factors that can counter the complement cascade of bacterial destruction. Pneumococcal surface proteins A and C (PspA and PspC), Pneumococcal histidine triad proteins (Phts), LytA (autolysin), Endopeptidase O (PepO), Enolase, and Tuf contribute to the inhibition of C3 convertase formation [16,17]. C3 convertase cleaves complement protein C3 into C3a and C3b, with C3a playing a crucial role in recruiting immune cells to destroy the invading bacteria and C3b playing a role in opsonization. The inhibition of C3 convertase causes fewer pathogens to be opsonized and fewer immune cells to be recruited to the site of infection, allowing pathogens to infect more areas [16,17,18]. In older individuals, this can have severe effects due to the fact that many older patients have existing health conditions such as diabetes or heart complications. The spread of infections in these individuals can drastically worsen preexisting conditions and lead to prolonged hospitalization, which may in turn increase the risk of hospital-acquired pneumococcal disease.

Pneumococcus can undergo phase variation between transparent and opaque phenotypes due to gene regulation of its capsule. In its transparent phenotype, *S. pneumoniae* has a thin capsule and has a high expression of phosphorylcholine (ChoP) and choline-binding protein A (CbpA), which allows colonization of the nasopharynx. In its opaque phenotype, on the other hand, *S. pneumoniae* has a thicker capsule and expresses pneumococcal surface protein A (PspA) that helps it survive in blood by reducing opsonization by binding to host proteins and complement factors [16,18,19,20,21,22]. The ability of *S. pneumoniae* to circulate in the blood can lead to sepsis and meningitis in severe cases and can greatly increase the risk of death, demonstrating that phase variation in *S. pneumoniae* can lead to evasion from immune responses due to the change in expression of surface proteins and capsule thickness, which can make it difficult for immune cells to properly identify *S. pneumoniae*. Coupled with reduced T cell production due to thymus shrinkage and reduced immune cell production in the bone marrow, older individuals often suffer from more severe symptoms since the lower count of immune cells makes it harder for their immune system to completely destroy the strain of *S. pneumoniae* and the phase variation can add a further obstacle for immune cells trying to identify and attack the bacterium. Additionally, *S. pneumoniae* can undergo mutations that can lead to a variety of different serotypes that can render some vaccines ineffective as it can lead to the formation of strains that are not accounted for by vaccines, making these individuals even more vulnerable to the effects of *S. pneumoniae* infection. Variability can also lead to prolonged symptoms from the immune system’s unfamiliarity with the serotype, which can lead to a delayed immune response. Diverse serotypes may also make some antibiotics ineffective as new serotypes may be more resistant to the medication [23].

## 3. Challenges with Diagnosis and Treatments

Common symptoms of *Streptococcus pneumoniae* infection include coughing, fever, chills, shortness of breath, chest pain, fatigue, abnormal breathing sounds, and respiratory distress. Chest X-rays are often conducted to check for infiltration and consolidation. X-rays are conducted to check for air in small airways replaced with fluid, a common indicator for strep pneumonia. CT scans can further demonstrate the condition of the lungs and sputum tests can be used to identify the specific bacteria causing the lung infection by taking a sample of lung fluid from a deep cough [24]. However, with common symptoms for pneumococcal disease being prevalent in other bacterial or viral infections, it is often challenging to diagnose older patients who often have underlying conditions with strep pneumonia due to the overlapping symptoms with other bacterial or viral infections such as a fever, cough, fatigue, shortness of breath, or chest pain [24]. Additionally, symptoms from an *S. pneumoniae* infection can sometimes be overshadowed by underlying chronic conditions such as heart failure, which may also cause symptoms like shortness of breath [24]. In addition, older individuals tend to have weaker immune systems that can lead to less apparent inflammatory responses and more mild symptoms until the more severe stages of infection, making it more difficult to treat. A weakened immune system can also lead to a more rapid onset of severe conditions that can put their lives at risk. Older patients with cognitive impairment may also struggle to communicate their symptoms, which makes it harder to diagnose these patients at early stages.

*S. pneumoniae* has over 100 known serotypes that, in some cases, can aid with pneumococcal disease diagnosis. Serotypes 1, 4, 5, 7F, 8, 12F, 14, 18C, and 19A are likely to cause invasive pneumococcal disease (IPD), a serious infection in which the *S. pneumoniae* invades areas of the body that are typically sterile, such as the bloodstream or central nervous system (CNS), with serotypes 1 and 19A being the most common serotype involved [25]. Serotype 14 commonly causes non-bacteremic pneumonia in adults, an infection localized in the lungs [24]. Serotypes 1, 3, and 19A are also associated with empyema and hemolytic uremic syndrome [25]. Urinary antigen detection (UAD) assays are common tests used to identify pneumococcal serotypes in urine samples. However, serotypes for pneumococcal disease tend to vary based on the age of the patient. Although serotypes 1, 5, 6A, 6B, 7F, 12F, 15B, 15C, 19A, 19F, and 23F serotypes found in all age groups, 5 serotypes are only found in children under 5, 20 serotypes in older patients are not found in children under 5, and 22 serotypes in younger patients not found in patients over 64 [26,27]. These varying trends in serotypes have led to the need for different vaccines for these age groups. Conjugate vaccines are used for young patients and involve the combination of a weak antigen with a strong antigen to create a stronger response to the weak antigen. On the other hand, polysaccharide vaccines used for older individuals involve the use of polysaccharides derived from capsules of bacteria to stimulate an immune response that leads to the generation of antibodies that recognize these specific polysaccharides found on specific serotypes. The presence of two different vaccine types can create gaps in vaccination coverage based on vaccine availability, which can lead to gaps in immunity as the transition between age-specific vaccinations can leave individuals more susceptible to the disease. Many individuals are not aware of the distinction between the PCV15 and PCV20 vaccines that are recommended by the CDC for children and adults up to the age of 50 versus the PCV13 and PPSV23 vaccines that are recommended for adults aged 65 and older. This may lead individuals over the age of 65 to be unaware of the need for revaccination, which can lead to more severe symptoms and a delayed immune response if the individual were to be infected by a serotype of *S. pneumoniae* that wasn’t covered by their previous vaccine, demonstrating the importance of educating the public about distinctions between pneumococcal disease vaccine and the importance of revaccination.

## 4. Age-Related Decline in Immunity Impacts Disease Susceptibility

The age of an individual greatly impacts the strength of immune responses to pathogens like *S. pneumoniae*. In newborns, the adaptive immune system favors tolerogenic responses due to the exposure to new environmental antigens and the need to identify new harmless antigens [28,29,30]. This makes it easy for pathogens to infiltrate their immune system and cause damage to cells and tissues. In addition, their T and B cells have limited function as they have yet to mature and develop fully, leading to a reliance on passive immunity from maternal antibodies, which makes them more susceptible to infections since passive immunity is dependent upon the specific antibodies transferred by the mother, meaning that new bacterial or viral strains can infiltrate the immune system due to the lack of maternal antibodies that recognize these new strains [28,29].

As an individual ages, memory T and B cells accumulate and increase in strength and responsiveness. However, the immune system eventually starts to weaken at around age 50, producing a U-shaped curve of infection susceptibility with age. One of the major factors contributing to the decline in immune system function is the decrease in the function of the thymus [31,32,33]. As an individual ages, decreases in the production of thymosin, thymopoietin, and corticosteroids lead to thymus shrinkage as it loses lymphoid tissue and is replaced by fatty tissue. Decreases in key thymic hormones are brought about by the declining function of endocrine glands, which are responsible for producing these hormones. With age, DNA damage can accumulate due to the shortening of telomeres that occurs each time a cell divides or the accumulation of genetic mutations that can impact the proliferative capabilities of stem cells [34]. The impairment of stem cell function, often worsened by oxidative stress due to a poor diet, can make it hard for the immune system to regenerate cells or replace damaged tissues or cells [35]. With many older patients experiencing other pre-existing health conditions, prolonged inflammation can decrease the function of endocrine glands by damaging their structure and reducing the production of crucial hormones that facilitate immune cell regeneration. This can lead to a decline in memory T and B cells, which makes it challenging for the patient’s immune system to identify and destroy pathogens.

The declining function of the thymus can contribute to the weakening of T cells due to the lack of T cell regeneration [36,37]. Immune cells often undergo oxidative stress over time as a result of free radicals being produced as a byproduct of metabolic processes and a sedentary lifestyle. A lack of physical exercise can reduce the body’s ability to produce endogenous antioxidants, which are needed to neutralize free radicals. Physical activity also improves blood circulation, which can help deliver nutrients and remove waste products. A sedentary lifestyle may also contribute to psychological stress that can increase cortisol levels and increase free radical production. Increased oxidative stress can impair T cell function by inhibiting key transcription factors like NFAT and NF-κB, thereby disrupting T cell signaling pathways [38,39]. Conversely, oxidative stress also promotes the production of pro-inflammatory cytokines by activating inflammatory transcription factors such as NF-κB and AP-1, which upregulate cytokine gene expression. This dual role of oxidative stress can drive T cells toward inflammatory phenotypes, such as Th1 and Th17 subsets, further perpetuating chronic inflammation [39,40,41]. The damage brought about by oxidative stress and chronic inflammation has detrimental effects to older patients since the decreased thymic production hinders the ability of the patient’s immune system to replace damaged cells, leading to the increased presence of impaired T cells that weaken the immune system’s ability to fight pathogens such as *S. pneumoniae*. With T cells playing a major role in the adaptive immune response by interacting with antigen-presenting cells, B cells, and infected cells, a decline in T cell function can drastically hinder the ability of the immune system to fight off pathogens, which may lead to the onset of severe conditions.

Aging also significantly contributes to declining dendritic cell (DC) functions [42,43]. Over time, oxidative stress leads to telomere shortening, which reduces the proliferative capacity of DCs. When telomeres shorten, a DNA damage response (DRR) is triggered that leads to senescence and apoptosis. Senescence in stem cells can lead to a decreased ability of an individual’s immune system to regenerate DCs [44]. Senescent cells also secrete pro-inflammatory cytokines like NF-kB and growth factors like VEGF and FGF to clear damaged cells and facilitate tissue repair. However, the accumulation of senescent cells and pro-inflammatory cytokines in older individuals can lead to chronic low-grade inflammation, a process known as inflammaging. Inflammaging can lead to DC exhaustion from overstimulation and lead to impaired antigen presentation and reduced maturation [42,43]. Reduced DC function can increase the risk of autoimmunity since DCs responsible for maintaining self-tolerance are unable to properly present self-antigens to regulatory T cells (Tregs) as a result of reduced phagocytic capacity. Declining DC function can also reduce the ability of pattern recognition receptors (PRRs) on DCs to detect pathogens or recognize signs of cell or tissue damage [43]. A reduction in DC maturation as a result of stem cell senescence with age can lead to lower surface expression of costimulatory molecules such as CD80, CD86, CD40, and MHC class II, which are needed for T cell activation. In the presence of pathogens, reduced T-cell activation can hinder the ability of the individual’s immune system to effectively destroy the pathogen, leading to potential cell, tissue, and organ damage.

Inflammaging can lead to an activation of DCs at homeostasis [45,46]. Our studies have shown that DCs from aged individuals exhibit heightened NF-κB activation and increased cytokine secretion at baseline [46]. The inflammatory cytokines released by these activated DCs can impact nearby airway epithelial cells, causing damage and compromising barrier integrity [47,48]. This weakened barrier increases the susceptibility of older individuals to infections, as pathogens can more easily invade the lungs. Pathogens like *S. pneumoniae* can, therefore, readily colonize the lungs, resulting in severe consequences such as lung damage and respiratory distress, including difficulty breathing. The shift towards an inflammatory phenotype in DCs can decrease the ability of lung cells to repair and regenerate. Additionally, cumulative exposure to environmental factors such as air pollution in older individuals can further impair DC function in the lungs. Air pollutants often generate reactive oxygen species (ROS) that damage the cell membrane, mitochondria, and endoplasmic reticulum in DCs that disrupt their normal function [49]. This can hinder the ability of DCs to destroy harmful particles that accumulate in the lungs, increasing the risk of pulmonary fibrosis and the formation of scar tissue in the lungs from irritation from the accumulation of particles in the lungs.

As noted by Agrawal and Gupta in 2017, effective antigen presentation by DCs is required for effective vaccine responses [43]. Not only do DCs present vaccine antigens to T cells and B cells to promote the generation memory T and B cells, but DCs also secrete cytokines like IL-12 that are crucial for directing the adaptive immune response to the vaccine antigen and strengthening the immune system against the antigen. Reduced DC function in older individuals can lead to a blunted immune response from DCs, T cells, and B cells, and this incomplete activation of key immune cells can lead to weaker responses to adjuvants that are common in some vaccines. The reduced ability of DCs to activate B cells can lead to weaker antibody responses that may render the vaccine ineffective at preparing the immune system to fight off the corresponding pathogen. This demonstrates the crucial role that DCs play in mediating immune responses against pathogens and generating effective responses to vaccine antigens.

Declining thymus function and DC production from oxidative stress and the shortening of telomeres can have profound impacts on an individual’s immune system by contributing to inflammaging and cell senescence. The reduced function of DCs limits the presentation of antigens specific to *S. pneumoniae,* which is needed to recruit immune cells to destroy the pathogen. This leads to a weak immune response and allows the bacterium to colonize areas such as the lungs, which can lead to dangerous health complications like difficulty in breathing. Reduced T cell function can further hinder the ability of the immune system to recognize and attack *S. pneumoniae,* leading to prolonged, or in some cases, chronic complications such as declining organ function due to excess inflammation. The cell and tissue damage brought about by *S. pneumoniae* from its colonization and encouragement of inflammation, which is worsened by its genetic variability and its ability to undergo phase changes, can exacerbate the declining thymus function and DC production in older individuals, leading to an increased decline in immune system function characteristic of accelerated immune system aging.

## 5. Conclusions and Future Directions

To combat the detrimental effects of *S. pneumoniae* on older individuals, it is crucial to continue developing stronger antibiotics and more effective vaccines. In addition, hospitals must focus on preventing the spread of pneumococcal disease by implementing greater protective measures for patients and employees such as ensuring adequate ventilation, having strict procedures for isolating infected patients, and encouraging frequent handwashing for patients.

In multivariate analyses of immune cell populations, levels of cytokines release, gene expression, and the composition of antibodies in pneumococcus isolates, Mohanty and colleagues in 2023 investigated the percentage of pneumococcal disease cases that involved strains that were resistant to penicillin, macrolide, fluoroquinolone, extended-spectrum cephalosporin (ESC), and tetracycline drug classes. They found that from 2011 to 2020, the estimated percentage of IPD and noninvasive pneumococcal disease cases that were resistant to at least one of the drug classes increased from 40.1% to 50.4% and 56.7% to 66.1%, respectively. Additionally, their results show that from 2011 to 2020, *S. pneumoniae* resistance to at least two of the drug classes for noninvasive pneumococcal disease patients increased from 29.5% to 39.9% with an average annual percentage change of +2.2% [50,51]. These results demonstrate an increasing trend of *S. pneumoniae’s* resistance to common antibiotics, highlighting a crucial need for improving antibiotics and creating more effective vaccines.

According to a 2023 study conducted by Kobayashi and colleagues, the PPSV23 (23-Valent Pneumococcal Polysaccharide Vaccine) vaccine has an estimated efficacy of only 63% in adults worldwide against IPD [52]. However, recent medical advancements have shown increases in the percentage of *S. pneumoniae* serotypes that can be covered by vaccines. In June of 2024, CAPVAXIVE (Pneumococcal 21-valent Conjugate Vaccine) was approved by the FDA for individuals over the age of 18 and covers about 84% of the most common invasive pneumococcal diseases, while PVC20 (Pneumococcal vaccine 20-valent Conjugate Vaccine), one of the most common pneumococcal disease vaccines, only accounts for about 52% [51]. This demonstrates that new vaccines are beginning to account for a larger percentage of serotypes of pneumococcal disease and may contribute to more effective immunity. Xacduro (Sulbactam-durlobactam), a new antibiotic approved by the FDA in May 2023, has been shown to reduce patient mortality by approximately 13.3% compared to Colistin (polymyxin E) [53]. However, *S. pneumoniae,* like many other bacteria, tends to adapt over time to avoid the effects of vaccines and antibiotics, revealing the need to continue allocating resources to improve treatments for patients infected with pneumococcal disease.

## Data Availability

No new data was created or analyzed in this study. Data sharing is not applicable to this article.
